# A Novel Shell Material—Highland Barley Starch for Microencapsulation of Cinnamon Essential Oil with Different Preparation Methods

**DOI:** 10.3390/ma13051192

**Published:** 2020-03-06

**Authors:** Liang Li, Wenhui Zhang, Jian Peng, Bei Xue, Zhendong Liu, Zhang Luo, Deze Lu, Xiaorui Zhao

**Affiliations:** 1 Food Science College, TAAHC-SWU Medicinal Plants Joint Research and Development Centre, Tibet Agriculture & Animal Husbandry University, Nyingchi 860000, China; jwllok@sina.com (L.L.); xuebei_1128@126.com (B.X.); ldzldz1998@sina.com (D.L.); zxrzx1996@sina.com (X.Z.); 2 Institute of Agriculture Products Development and Food Science Research, Tibet Academy of Agriculture and Animal Science, Lhasa 850032, China; zhhf08@163.com; 3 Sericultural & Agri-Food Research Institute, Guangdong Academy of Agricultural Sciences, Guangzhou 510610, China; pengjian@gdaas.cn

**Keywords:** Microcapsules, Highland barley starch, molecular inclusion method

## Abstract

Highland barley starch (HBS), as a carbohydrate shell material with excellent performance in microcapsule applications, has rarely been reported. In the present study, three different microcapsules (CEO-SWSM, CEO-PM, and CEO-UM) were synthesized successfully via saturated aqueous solution method, molecular inclusion method and ultrasonic method, respectively, using HBS as shell material coupled with cinnamon essential oil (CEO) as the core material. The potential of HBS as a new shell material and the influence of synthetic methods on the performance of microcapsules, encapsulation efficiency (EE), yield, and release rate of CEO-SWSM, CEO-PM, and CEO-UM were determined, respectively. The results confirmed that CEO-PM had the most excellent EE (88.2%), yield (79.1%), as well as lowest release rate (11.5%, after 25 days of storage). Moreover, different kinetic models were applied to fit the release process of these three kinds of microcapsules: CEO-SWSM, CEO-PM, and CEO-UM had the uppermost R-squared value in the Higuchi model, the zero-order model, and the first-level model, respectively. Over all, this work put forward a novel perspective for the improved encapsulation effect of perishable core materials (e.g., essential oil) for the food industry.

## 1. Introduction

Cinnamon (Cinnamomum cassia Presl) is a natural plant widely cultivated in tropical and subtropical regions [1]. In general, we could extract cinnamon essential oil (CEO), a yellow or amber liquid with a peculiar sweet aroma, from the branches, leaves, fruits, and pedicels of cinnamon, and the main components of it is cinnamaldehyde (>98%) [2,3]. In recent years, its excellent antibacterial properties (for bacteria, fungus and mucedine) had received increasing attention, meanwhile, it was also an efficient and healthy antioxidant and stabilizer of foodstuff [4,5]. However, when the active ingredient in CEO was affected by external factors such as air, light, moisture, and temperature, it would accelerate evaporation or even inactivation [3,6].

Fortunately, microcapsule technology was very good at protecting core materials’ (e.g., essential oil) volatility and perishability from external factors [7,8,9]. Therefore, CEO could be extensively used in the food industry by microcapsulary, which could restrict its inactivation or volatilization during processing and storage [10,11]. In general, the synthesis methods and the types of shell materials were the main factors affecting the packaging effect of microcapsules [12,13].

For synthesis methods, it can be roughly divided into two major categories: physical methods and chemical methods. Concretely, common physical methods include kneading, saturated aqueous solution, molecular inclusion, freeze-drying, ultrasonic, and spray-drying. The operation steps of the kneading method [14], the saturated aqueous solution method [13], and the ultrasonic method [15] were operated easily, while the encapsulation efficiency (EE) of the synthesized microcapsules was greatly limited. The freeze-drying method [16] and the spray-drying method [17] had higher EE, but the operation steps were cumbersome, and peculiar equipment was needed. It is worth noting that the molecular inclusion method [18,19] had the above two advantages. In addition to physical methods, there are many reports on the synthesis of microcapsules by chemical methods, such as electrodeposition method [20], emulsion template method [21,22], and photo cross-linking method [23]. Since most of the chemical methods synthesized microcapsules had the disadvantages, including a small amount of chemical residues (high toxicity) and complicated operation steps, they were replaced by physical methods gradually.

For the shell materials, it also can be roughly divided into two categories: natural materials and artificial materials. Among them, artificial materials were gradually replaced by natural materials due to their complicated preparation process and high toxicity [24,25,26]. Natural materials can be subdivided into proteins and carbohydrates. On account of the rich structure of functional groups (hydrophilic and hydrophobic) of protein surface, they could play the role of emulsifiers in the emulsion heat-induced cross-linking systems [27], which greatly improved the EE of microcapsules. But at the same time, unfortunately, due to some instability of their own, co-stabilizers were often needed to introduce into these systems to complete the entire crosslinking process [28,29]. Therefore, a small amount of co-stabilizer was easily retained in the synthesized protein-based microcapsules, which would limit the application of microcapsules in a certain degree. Additionally, carbohydrate-based materials such as starch [30,31,32], maltodextrin [33,34], and chitosan [35,36] also could be used as high-quality shell materials for embedding hydrophobic core materials. 

Highland barley was mainly distributed in the Qinghai-Tibet Plateau as an important grain crop in Tibet, and widely used in the food industry, such as alcoholic beverages and cooked wheaten food products [37,38]. Highland barley had rich nutritional components including high protein, high fiber, high vitamin, low fat, and low sugar, and it was rich in β-glucose, which had a significant effect on preventing cardiovascular diseases and diabetes [39,40]. Starch is the main functional component of highland barley; occupying 75% to 80% of in the endosperm [41]. As the previous research reported, there are several advantages of highland barley starch (HBS), such as good film forming performance, low immunogenicity, outstanding freeze-thaw stability, high solubility, excellent emulsifying stability, low viscosity, favorable stability, good biocompatibility, biodegradable, non-toxic side effects, and so on [41,42,43]. These superiorities will contribute to embedding core materials and retaining their physicochemical properties. However, up to now, there have been few reports on the use of HBS as a shell material in microcapsules.

Here, we successfully synthesized three different microcapsules (CEO-SWSM, CEO-PM, and CEO-UM) via saturated aqueous solution method, molecular inclusion method, and ultrasonic method, respectively, in which HBS as shell material and CEO as core material. Then, in order to understand the potential of HBS as a new shell material, and to obtain the most suitable preparation method for this microcapsule system, we evaluated the embedding performance of HBS by detecting the EE, yield and release rate of CEO-SWSM, CEO-PM, and CEO-UM, respectively. Furthermore, the zero-order kinetic model, the first-order kinetic model, and the Higuchi kinetic model were used to fit the release process of three types of microcapsules, the release rate constants, and the R-squared values of the microcapsules were obtained and the probable release mechanisms of the microcapsules proposed. On the one hand, this work provides a novel shell material for microcapsule of CEO; on the other hand, it provides a basis for microcapsules to more effectively control the release of core materials.

## 2. Materials and Methods 

### 2.1. Materials

A CEO was purchased from Alfa Aesar Chemical Co., Ltd. (Heysham, UK). HBS was purchased from Tibet Academy of Agriculture and Animal Sciences (TAAAS, Zangqing 2000, Lhasa, China). Other reagents were purchased from Sinopharm Chemical Reagent Co., Ltd. (Shanghai, China). All of the reagents were used as received without further purification.

### 2.2. Synthesis of Microcapsules

#### 2.2.1. Saturated Aqueous Solution Method

**Scheme 1:**
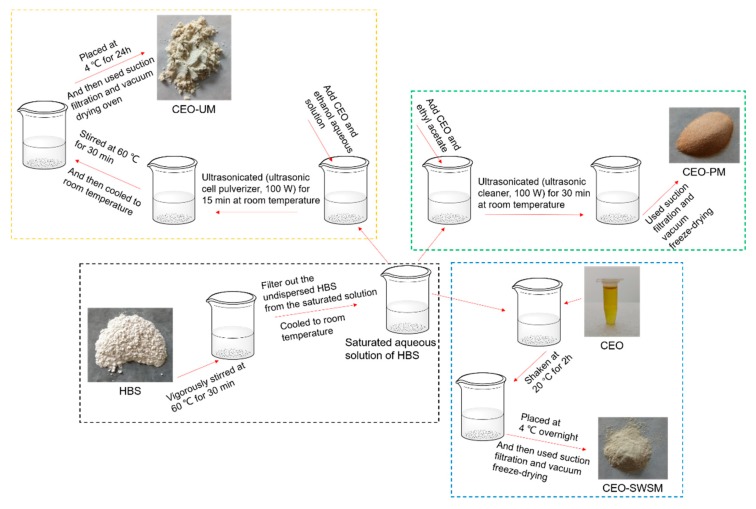
Schematic of the preparation process to form CEO-SWSM, CEO-PM and CEO-UM.

As shown in Scheme 1, firstly, excess HBS was slowly added to a 250 mL beaker containing deionized water, after being vigorously stirred at 60 °C for 30 min with a magnetic heating stirrer (Gongyi Yuhua Instrument Co., Ltd., Gongyi, China), cooled to room temperature, and the undispersed HBS in the saturated solution was filtered off. Secondly, the CEO was added into the pre-prepared saturated aqueous solution in a mass ratio of CEO and HBS 1:5, and shaken in an orbital shaker incubator (Shanghai Xinyi Instrument Co., Ltd., Shanghai, China) at 20 °C for 2 h. Tertiary, the mixture was stored in a refrigerator (Guangzhou Anfei Environmental Protection Technology Co., Ltd., Guangzhou, China) at 4 °C overnight, and then filtrated (Gongyi Yuhua Instrument Co., Ltd.) and vacuum freeze-drying (Beijing Biocool experimental instrument co. Ltd., Gongyi, China) to finally obtain pale yellow sample.

#### 2.2.2. Molecular Inclusion Method

As shown in Scheme 1, firstly, configured ethyl acetate and CEO as a mixed solution in a volume ratio of 5:1, and added to the pre-prepared saturated aqueous solution of HBS according to the mass ratio of CEO and HBS of 1:5. Secondly, the mixture was ultrasonicated (ultrasonic cleaner, Kunshan Ultrasonic Instrument Co., Ltd., Kunshan, China, 100 W, 25 °C) for 30 min at room temperature. Tertiary, filtrated (Gongyi Yuhua Instrument Co., Ltd.) and vacuum freeze-drying (Beijing Biocool experimental instrument co. Ltd.) to finally obtain light orange sample.

#### 2.2.3. Ultrasonic Method

As shown in Scheme 1, firstly, added a certain amount of CEO to an ethanol aqueous solution with volume concentration of 60% and stir until dissolved. Secondly, the CEO was added into the pre-prepared saturated aqueous solution in a mass ratio of CEO and HBS 1:5, and placed a 250 mL beaker containing the above mixed solution into an ultrasonic cell pulverizer (Kunshan Ultrasonic Instrument Co., Ltd., 100 W, 25 °C) and ultrasonicated for 15 min at room temperature. Subsequently, we stirred the mixed solution in a water bath (Gongyi Yuhua Instrument Co., Ltd.) at 60 °C for 30 min. Tertiary, after the mixture was naturally cooled to room temperature, it was placed in an explosion-proof refrigerator (Guangzhou Anfei Environmental Protection Technology Co., Ltd.) at a temperature of 4 °C for 24 h. And then filtrated (Gongyi Yuhua Instrument Co., Ltd.) and vacuum dried (Wuxi Marite Technology Co., Ltd., Wuxi, China) with temperature set to 40 °C to finally obtain a dark yellow microcapsule powder.

### 2.3. Characterizations

Powder X-ray diffraction (XRD) patterns of as-prepared samples were conducted with a diffractometer (Bruker D8 ADVANCE, Karlsruhe, Germany) equipped with a Cu-Kα radiation source operated at 40 kV and 30 mA. Fourier transform infrared (FT-IR) spectra were recorded using a spectrometer (Nicolet IS10, Waltham, MA, USA) within the wavenumber range of 400 to 4000 cm^−1^ with a resolution of 2 cm^−1^ and using KBr as the beam splitter, and liquid samples could be tested by painting between two plates of potassium bromide. The morphology of the as-prepared samples was observed by field-emission scanning electron microscopy (FE-SEM, SU8010, Tokyo, Japan). The size distributions and Zeta-potential values of samples were conducted through a dynamic light scattering (DLS, Malvern Zeta-sizer Nano-S, Malvern, UK), and every sample was measured at least three times. Differential scanning calorimeter (DSC) of the as-prepared samples was conducted with a simultaneous thermal analyzer (MEITTLER STA449F3, Selb, Germany) used an empty crucible as a reference (scanning temperature range was 25~250 °C; heating rate was 10 °C/min; nitrogen flow rate was 100 mL/min). Nuclear magnetic resonance hydrogen (^1^H NMR) spectrum were measured with an NMR spectrometer (Bruker AVANCE III HD, 400MHz). Each sample was prepared by methanol-D.

### 2.4. Performance

#### 2.4.1. Surface Oil Content

Appropriate amounts of microcapsule particles were added to 10 mL of petroleum ether and continued to stir for 5 min at room temperature. Subsequently, the suspension was obtained by filtration, and the residue was repeatedly washed three times with 5 mL of petroleum ether. Then, the suspension and washings were evaporated under reduced pressure at 50 °C. The surface oil content was calculated as the difference in weight of the flask before and after the evaporation experiment. The tests were conducted in triplicates.

#### 2.4.2. Total Oil Content

The total oil content was determined by alkaline diethyl ether extraction. Specifically, first, we added 1.25 mL of aqueous ammonia to a 10 mL sample (1 mg) dispersion, well mixed and added 10 mL of ethanol, shaken well and kept in a 60 °C water bath for 5 min. After cooled to room temperature, it was transferred to a separating funnel and 25 mL of anhydrous diethyl ether was added, and extracted repeatedly for 2 to 3 times, and then the collected organic layer was placed in a flask to evaporate all the solvent under reduced pressure. The total oil content was calculated by the difference in weight of the flask before and after the evaporation experiment. The result was the average of three measurements repeated for each sample.

#### 2.4.3. Encapsulation Efficiency

EE (%) of the microcapsules, which determined the weight of oil embedded in the HBS, was calculated with Formula (1):

*EE* = (*O_t_* − *O_s_*)/*O_t_* ∗ 100%, (1)

where *O_t_* and *O_s_* were the weight of the total oil and the surface oil in the microcapsules, respectively [12].

#### 2.4.4. Microcapsule Yield

Microcapsule yield (MY, %), which determined the quality of the microcapsules, was calculated as Formula (2):

*MY* = *M_a_/M_t_* ∗ 100%, (2)

where *M_t_* was the theoretical quality of the microcapsules and Ma was the actual quality of the microcapsules [13].

#### 2.4.5. Slow Release Performance of Microcapsules and Their Release Kinetic Fitting Models

The microcapsules, which encapsulated the CEO, were stored at a room temperature of 15 ± 5 °C with a relative humidity of 60 ± 10% in darkness for 25 days. During this period, samples were analyzed every 5 days using soxhlet extraction method. The release rate (RR) of the microcapsule was calculated according to Formula (3).

*RR* = (1 − *M_to_*)/*M_o_* ∗ 100%, (3)

where *M_to_* was the quality of CEO in the microcapsules after storage for a period of time and Mo was the initial mass of CEO in the microcapsules.

The zero-order kinetic model: *RR* = *a* + *bt*, the first-order kinetic model: ln (1 − *RR*) = *a* + *bt* and the Higuchi kinetic model: *RR* = *bt*^1/2^ (where *RR* (%) was the release rate of CEO in the microcapsule; t(d) was the microcapsule storage time; *a*, *b* were the formula parameters) were used to fit the release kinetic models of CEO-SWSM, CEO-PM, and CEO-UM, respectively.

### 2.5. Statistical Analysis

The data were expressed as the mean ± standard deviation and one-way analysis of variance was conducted by Duncan test using SPSS Statistics Version 19 (IBM Corporation, Chicago, IL, USA).

## 3. Results and Discussion

### 3.1. Characterization of Microcapsules

#### 3.1.1. Powder XRD

**Figure 1:**
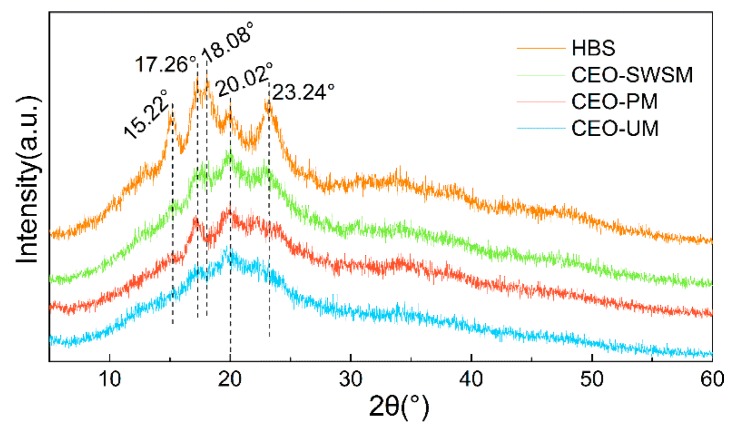
X-ray diffraction (XRD) patterns of highland barley starch (HBS) and the CEO-SWSM, CEO-PM, and CEO-UM microcapsules.

As shown in Figure 1, both the diffraction peak and the diffraction dispersion appeared in the diffraction pattern of HBS, which indicated that the internal structure of HBS was composed of the crystalline region (A-type crystalline structure) and the amorphous region (V-type crystalline structure), and the complex interaction between the double-helical structure of amylopectin was the main reason for the formation of the A-type crystalline structure of HBS [42]. Concretely, the peak of HBS at 15.22°, 17.26°, 18.08°, and 23.24° were all attributed to the double-helical structure of amylopectin, and the amorphous diffraction peak near 20.02° was attributed to amylose [44]. In the pattern of CEO-SWSM, CEO-PM, and CEO-UM, all diffraction peaks belong to HBS and the intensity of all diffraction peaks belonging to the A-type crystalline structure of HBS had decreased to various degrees, among them, CEO-UM was the most obvious. However, the intensity of the V-type peak near 20.2° in the three kinds of microcapsules did not decrease significantly, even when it became the main diffraction peak in their diffraction patterns. Above all, it can be concluded that CEO selectively covered on the surface of amylopectin may be the main reason for the weakening of the diffraction peak intensity in the A-type crystalline structure of HBS, and this provided the possibility for HBS as a good packaging material for the CEO.

#### 3.1.2. FT-IR

**Figure 2:**
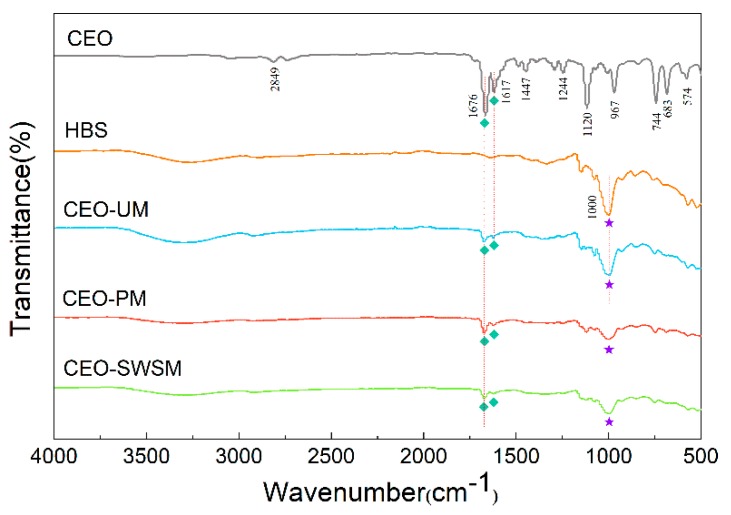
FT-IR spectra of CEO, HBS, and the CEO-SWSM, CEO-PM, and CEO-UM microcapsules.

The valence structures of CEO, HBS, and CEO-SWSM, CEO-PM, CEO-UM were determined by FT-IR spectroscopy. As shown in Figure 2, the characteristic peak at 2849 cm^−1^ was attributed to the overlapping symmetric and asymmetric stretching vibration of C-H bond [45]; the two typical peaks at 1676 cm^−1^ and 1617 cm^−1^ were consistent with the stretching vibration peak of carbonyl and unsaturated vibration peak of benzene ring, respectively [46]; the characteristic peak located at 1244 cm^-1^ was the lactones stretching vibration peak of C-O bond [47]. The main components in CEO were cinnamon aldehyde, α-phellandene, 1,8-eucalyptol, etc. The basic structures constituting these organic components were aromatic rings and alicyclic rings, and some functional groups such as the carboxyl group, the hydroxyl group, the carbonyl group, and the methoxy group. It is worth noting that both stretching vibration peak of carbonyl and unsaturated vibration peak of benzene ring of CEO could be markedly observed in the FT-IR spectra of CEO-SWSM, CEO-PM, and CEO-UM, this result confirmed the presence of CEO in these three kinds of microcapsules. In addition, we found that the characteristic peak located at 1000 cm^−1^ in the FT-IR spectrum of HBS could also be viewed in the spectra of three kinds of microcapsules, this result proved the existence of HBS in microcapsules in a further step.

#### 3.1.3. DLS

**Figure 3:**
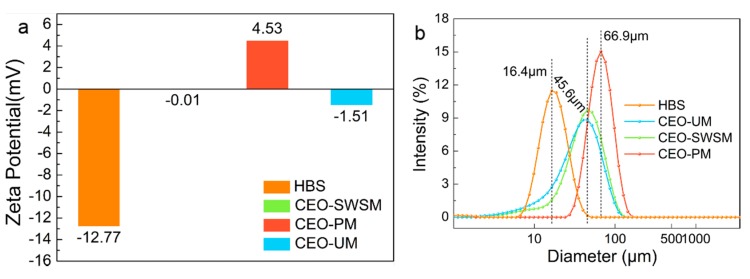
(**a**) Zeta-potential values and (**b**) size distributions of HBS and the CEO-SWSM, CEO-PM, and CEO-UM microcapsules determined by DLS.

The Zeta-potential values and size distributions of HBS and CEO-SWSM, CEO-PM, CEO-UM were analyzed using DLS [48,49]. As shown in Figure 3a, the Zeta-potential values of these three kinds of microcapsules were increased in various degrees when compared with HBS, especially for CEO-PM (4.53 mV). The positively charged carbonyl in cinnamaldehyde [1], which was the main component of CEO, was easily attracted by the negative charge on HBS that caused some of the negative charge to be neutralized. As the mass of CEO increased, the potential of the microcapsules would be reversed from negative to positive. Furthermore, among the three kinds of microcapsules, the highest Zeta-potential value was obtained from CEO-PM, in other words, it had the best stability [50].

Size distributions of the HBS and microcapsules are presented in Figure 3b. HBS has an average diameter of 16.4 μm, when HBS was prepared into three kinds of microcapsules through different packaging methods, their average diameters were increased significantly. CEO-PM has the largest average diameter (66.9 μm), and the most uniform size distribution. Besides, the average diameters of CEO-UM and CEO-SWSM were similar, and the CEO-SWSM was slightly larger than CEO-UM. The average diameters of the microcapsules might be related to the quality of the CEO embedded in the HBS, that is to say, the average diameters of the three kinds of microcapsules was positively correlated with their EE.

#### 3.1.4. FE-SEM

**Figure 4:**
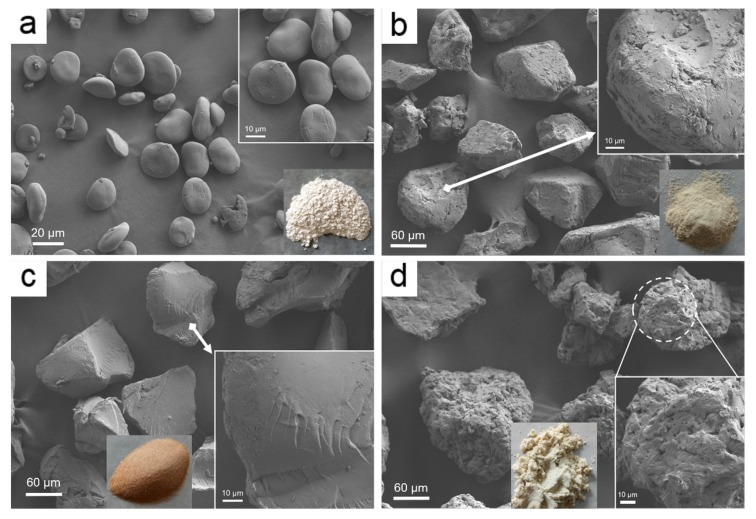
FE-SEM images of (**a**) HBS, (**b**) CEO-SWSM, (**c**) CEO-PM, and (**d**) CEO-UM microcapsules.

FE-SEM images of the HBS and CEO-SWSM, CEO-PM, CEO-UM microcapsules were displayed in Figure 4. The pristine HBS in Figure 4a showed that it consists of irregular, smooth-surfaced microparticles, which were almost interconnected with the diameter of 15–30 μm, and some of the HBS were ellipsoid [41]. Compared to the HBS, the three kinds of microcapsules, CEO-SWSM, CEO-PM, and CEO-UM, had significantly increased in diameter, this was consistent with the result obtained by DLS. Specifically, according to Figure 4b and its enlarged images, we discovered that the surface of the CEO-SWSM was distributed with many irregular nanoparticles of different sizes, making its surface appear rough. Combined with the EE of CEO-SWSM, it could be inferred that these nanoparticles distributed on its surface were unencapsulated CEOs. Additionally, Figure 4d and its enlarged images showed that the CEO-UM exhibited a porous structure with a large number of nanoparticles on its surface, similarly, these nanoparticles were also unencapsulated CEOs. The porous structure might be the main reason of the low storage performance of CEO-UM for CEO. However, we detected that CEO-PM was different in structure from the other two microcapsules, and its surface was smoother and denser than the CEO-SWSM and CEO-UM (Figure 4c and its enlarged images), and no obvious roughs were observed. This structure was a good explanation of the high EE of CEO-PM for CEO and its excellent storage performance.

#### 3.1.5. DSC

**Figure 5:**
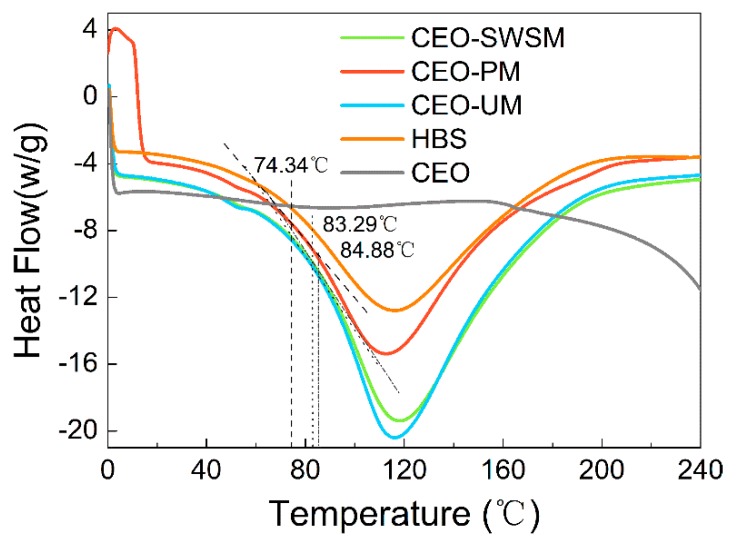
Differential scanning calorimeter (DSC) heating thermograms of pure HBS, CEO, and the microcapsules synthesized via saturated aqueous solution method (CEO-SWSM), molecular inclusion method (CEO-PM), and ultrasonic method (CEO-UM).

To investigate the thermal stability of the samples, the glass transition temperatures (T_g_) of these microcapsules were analyzed using the DSC. As shown in Figure 5, the heat flow of HBS and CEO-SWSM, CEO-PM, CEO-UM remained essentially unchanged before and after the differential scanning, which indicated that HBS had excellent thermal stability and was suitable as a shell material of microcapsules [51]. The phase change latent heat of the three kinds of microcapsules, which embedded with CEO, was significantly lower than that of pure CEO, and the low core/shell mass ratio was the main reason that was responsible for this result [52]. In addition, T_g_ of CEO-SWSM, CEO-PM, and CEO-UM were 84.88 °C, 74.34 °C, and 83.29 °C, respectively, which were much higher than the common storage temperature. This means that the three kinds of microcapsules maintained high stability in most storage environments. As the temperature continued to rise and was greater than T_g_, the molecular chain of HBS began to thaw, and the structure of the shell material was gradually destroyed. When the temperature reached T_m_, the swelling and softening shell material would not effectively encapsulate the CEO [53]. The T_m_ of the three kinds of microcapsules were 118.33 °C, 113.17 °C, and 116.33 °C, respectively (as shown in Figure 5), which could satisfy the requirements of the majority of high-temperature operation in the food industry, and expanded the application of the microcapsules.

### 3.2. Performance of Microcapsule

#### 3.2.1. Encapsulation Efficiency and Yield of Microcapsule

**Figure 6:**
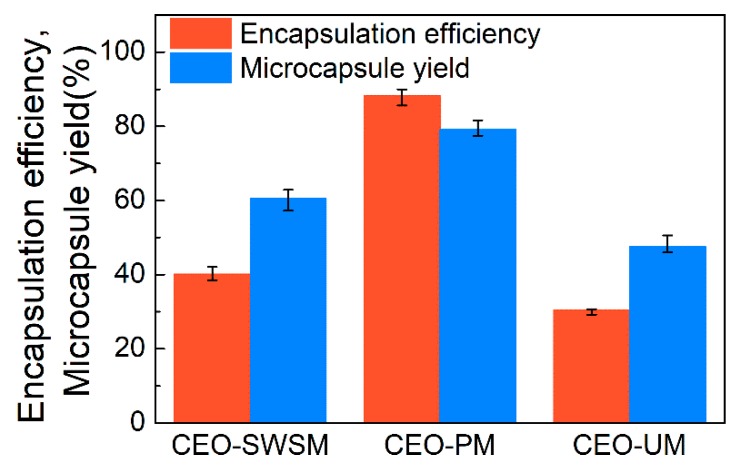
Encapsulation efficiencies and microcapsule yields of CEO-SWSM, CEO-PM, and CEO-UM.

As shown in Figure 6, there is a significant difference in the yield of the three microcapsules, concretely, CEO-PM, CEO-SWSM, and CEO-UM were 79.1%, 60.5%, and 47.5%, respectively. The encapsulation efficiency (EE) of CEO-PM was higher than the other two microcapsules, the EE of CEO-PM, CEO-SWSM, and CEO-UM was 88.2%, 40.1%, and 30.4%, respectively. This is owed to the ultrasonic cavitation and the appropriate embedding temperature. This result validated the inferences in DLS and FE-SEM. Additionally, the EE of the three kinds of microcapsules increased with increasing their yield, this result indicated that whether using saturated aqueous solution method, molecular inclusion method, or ultrasonic method, HBS was always more inclined to play the role of shell material, embedding CEO inside. Subsequently, as shown in Table 1, we compared the performance of CEO-PM to microcapsules synthesized with other shell materials.

#### 3.2.2. Slow Release Performance of Microcapsules

**Figure 7:**
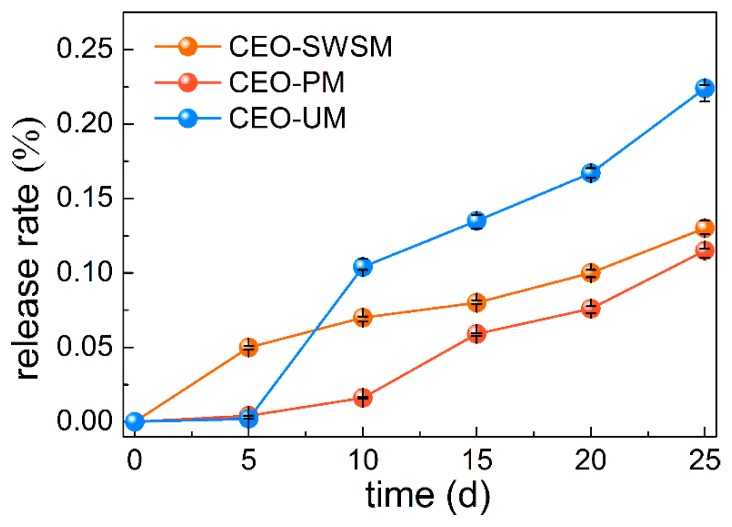
Release rates of the microcapsules synthesized by saturated aqueous solution method (CEO-SWSM), molecular inclusion method (CEO-PM), and ultrasonic method (CEO-UM).

In order to evaluate the slow release performance of microcapsules obtained by three different preparation methods, release tests of the CEO were carried out [13]. As shown in Figure 7, the release rates of the three kinds of microcapsules gradually raised with the extension of the storage days. Concretely, CEO-UM had the fastest release rate, that is to say, the microcapsule synthesized by ultrasonic method had poor slow release property, and loose porous structure might be the main cause for this result (as shown in Figure 4d). It is worth noting that the release rate of CEO-PM was always lower than CEO-SWSM and CEO-UM, and after 25 days of storage, the release rate of encapsulated CEO was only 11.5%, much smaller than 22.4% of CEO-UM. From this we concluded that the excellent slow release property of CEO-PM was closely related to its dense and smooth microstructure, that is to say, the microcapsules produced using molecular inclusion method retained good solid skeleton [54], so the density of the shell material-HBS was stronger than that of the microcapsule prepared by ultrasonic method. In addition, although CEO-SWSM also had a lower release rate, its EE was only half that of CEO-PM.

**Figure 8:**
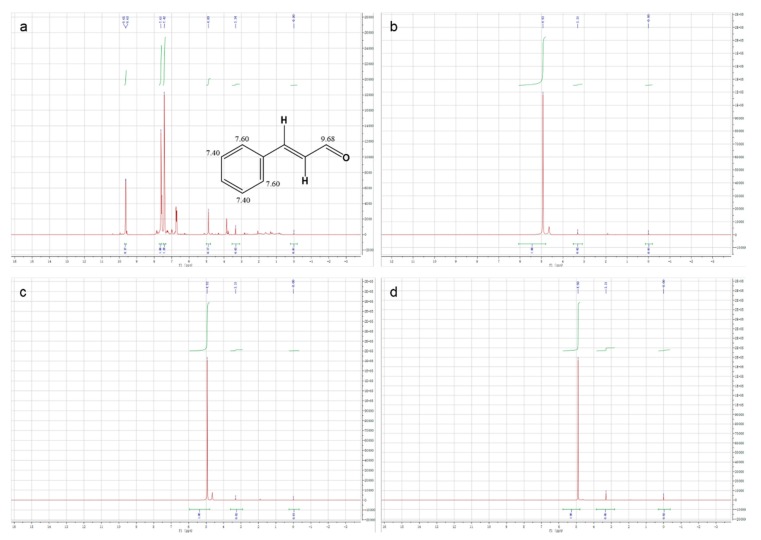
1 H NMR spectra of (**a**) CEO, (**b**) CEO-SWSM, (**c**) CEO-PM, and (**d**) CEO-UM microcapsules.

On the other hand, in order to more objectively reflect the excellent embedding performance of HBS as a shell material, we performed ^1^H NMR on the methanol-D, which soaked the microcapsules. In detail, we placed three kinds of microcapsules dissolved in methanol-D for 3 days. To ensure the authenticity, the surface oil of the microcapsules was removed. In ^1^H NMR spectrum of pure CEO (as shown in Figure 8a), the characteristic peaks attributed to cinnamaldehyde, the main component of CEO, could be clearly observed. Therein, the characteristic peaks at 9.63 ppm and 9.65 ppm were attributed to hydrogen on the carbonyl carbon in cinnamaldehyde; two different peaks were situated at 7.42 ppm and 7.61 ppm, and they were consistent with the hydrogen on the benzene ring carbon. Besides, the ^1^H NMR spectrum of CEO also showed a solvent peak at 3.34 ppm attributed to methanol-D and an impurity peak at 4.89 ppm attributed to residual pure water in CEO. Then in the ^1^H NMR spectrum of methanol-D, which soaked the microcapsules for 3 days (as shown in Figure 8b-d), only the impurity peaks belonging to pure water were observed, which indicated that the CEO was encapsulated in the microcapsules by HBS without any leak, that is to say, HBS was an ideal shell material.

In summary, among the three kinds of microcapsules, the CEO-PM synthesized by molecular inclusion method had the best slow release property and EE.

#### 3.2.3. Release Kinetics of Microcapsules

To better understand the release process of the three kinds of microcapsules, three fitting models were applied to simulate the release kinetic characteristics of CEO-SWSM, CEO-PM, and CEO-UM, respectively.

**Figure 9:**
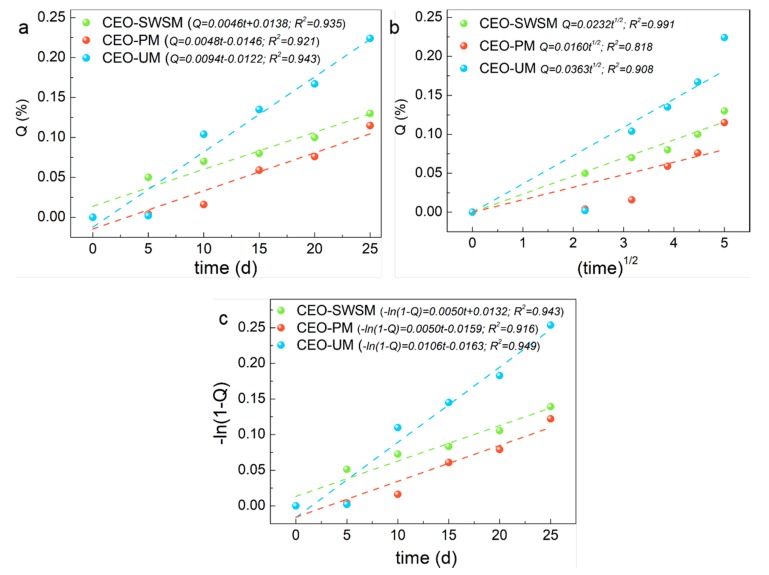
Release kinetics fitting equations of the CEO-SWSM, CEO-PM, and CEO-UM: (**a**) zero-order kinetic model, (**b**) Higuchi kinetic model, and (**c**) first-order kinetic model.

As shown in Figure 9, compared with the three kinds of release kinetic equations, CEO-PM had the highest R-squared value (0.921) in the zero-order kinetic equation (Figure 9a), that was to say, the release process of CEO-PM was more in line with the constant velocity diffusion release mechanism [55]. Specifically, during the release process of the CEO-PM, the release environment volume far outweighed the CEO-PM volume, and the size and surface area of it remained almost unchanged, at the same time, the mass of CEO in the CEO-PM was high, so the concentration of CEO in the CEO-PM might be approximately remained the same. Therefore, CEO was a steady-state diffusion in the membrane of the CEO-PM, which is an ideal diffusion process [56]. It provides better protection for CEO and greatly improves the stability of CEO.

Similarly, CEO-SWSM (0.991) and CEO-UM (0.949) had the maximum R-squared value in the Higuchi kinetic equation (Figure 9b) and the first-order kinetic equation (Figure 9c), respectively [57,58]. The release process of CEO-SWSM was most consistent with the Higuchi kinetic model, and the best kinetic model for the release process of CEO-UM was the first-order kinetic model. This was because the mass of CEO in the microcapsules prepared by the saturated aqueous solution method and the ultrasonic method were lower, and with the core materials were continuously released, the concentration of CEO in the microcapsules gradually decreased, and the release driving forces were continuously reduced, and then caused decline of the release rate [59]. Meanwhile, the main resistance of the release process of CEO-SWSM and CEO-UM were the density of the shell material, by reason of the saturated aqueous solution method retained the good solid skeleton, so the density of the shell material was stronger than that of the ultrasonic method [60]. Therefore, its protection against CEO was slightly stronger than the latter. Besides, the release rate constants of microcapsules produced using saturated aqueous solution method, molecular inclusion method and ultrasonic method were 0.0232 /d1/2, 0.0048 /d, 0.0106/d, respectively.

## 4. Conclusions

In this study, HBS as the shell material, was used to prepare CEO-filled microcapsules through three different methods. Among them, CEO-PM had the most outstanding embedding efficiency and the lowest release rate during storage, followed by CEO-SWSM and CEO-UM. According to the FESEM diagrams, it was confirmed that the excellent embedding efficiency and slow release performance of CEO-PM were closely related to its dense and smooth microstructure. In addition, ^1^H NMR also confirmed that HBS had an excellent protective effect on CEO. Thereafter, by fitting the release kinetic models of the three kinds of microcapsules, we found that the CEO-SWSM had the highest R-squared value (0.991) to the Higuchi kinetic equation, the CEO-PM had the highest R-squared value (0.921) to the zero-order kinetic equation, and CEO-UM had the highest R-squared value (0.949) to the first-order kinetic equation, which indicated that the CEO-PM release mechanism was an ideal steady-state process; the release mechanism of CEO-SWSM and CEO-UM was a diffusion process with a concentration-limiting step, this process was not conducive to the protection of the core material. In conclusion, HBS indeed had enormous potential as a shell material for microcapsules, and molecular embedding method was an economical and efficient preparation method for essential oil microcapsules.

## Figures and Tables

**Table 1:** The properties of microcapsules prepared with HBS were compared with those prepared with other shell materials.

Shell Material	EE	MY
β-cyclodextrin [12]	77.92%	87.99%
Jackfruit seed starch [12]	79.33%	84.82%
Chitosan [12]	76.64%	86.35%
Whey protein [28]	86.1%	-
This work	88.2%	79.1%
